# Supplementary material for: Are carbon emissions trading and green financial instruments synergistic? -Comprehensive quantitative research based on content analysis
Source: PLoS One. 2024 Mar 7;19(3):e0298601. doi: 10.1371/journal.pone.0298601 (PMC10919601; doi:10.1371/journal.pone.0298601)
Supplement: S1 Data — (ZIP) [file pone.0298601.s001.zip › Data/地级市Z_F工作报告.zip!/╝¬┴╓/═¿╗»╩╨/└┤╫╘╣1⁄2╓┌║┼í╛┬φ┐╦╩2╛▌═°í┐_2018.docx]

各位代表：

　　我代表市政府，向大会报告工作，请予审议，并请市政协各位委员提出意见。

　　一、2017年工作回顾2017年，是极不平凡的一年，各种困难和矛盾相互交织、集中显现。面对严峻的形势和繁重的任务，市政府在省委、省政府和市委的坚强领导下，在市人大、市政协的监督支持下，团结带领全市人民，主动作为、攻坚克难，全力做好稳增长、促改革、调结构、惠民生、防风险各项工作，经济社会健康发展。预计，实现地区生产总值900亿元、规模以上工业总产值1087.2亿元、地方级财政收入61.9亿元、社会消费品零售总额429亿元，完成固定资产投资696亿元。

　　（一）经济保持平稳运行。制定落实“国14条”80条具体措施，着力破解发展难题。滚动实施工业扩能提升工程，119个工业项目列入全省转型升级重大项目，18种新产品实现规模化生产，15户企业完成并购重组，金融机构本外币贷款余额增长10.9%，工业用电量增长6.3%，铁路货运量增长10.8%。支持通钢去产能、去杠杆，通钢列入全省债转股试点，5年来首次实现扭亏为盈。建材、机械制造、化工、能源等产业平稳健康发展。我市被评为全国消费品工业“三品”战略示范城市。扩大有效投资，开展第二个“冬季行动”、春季“三早”行动和重点项目观摩推进活动，落实项目清单管理和市级领导包保责任制，全市开复工5000万元以上项目342项，其中亿元以上项目268项。万通40亿片感通片、东宝胰岛素四期、修正医药科技产业园主体工程等一批重点项目竣工。全市累计向上争取资金233亿元。实施三年项目滚动计划，建立“一个千亿级、双十百亿级”重大项目储备库。主动“走出去”、积极“请进来”，招商引资成果丰硕，新纳入省“调度系统”项目105项，项目履约率达到92.4%，东方红西洋参产业基地、君必达中药制药产业园等一批项目相继落地。建立市级领导联系服务重点民营企业制度，出台促进民营企业发展18条政策举措，民营经济增加值占GDP 比重达到53.8%。（二）转型升级成效显著。启动大健康产业5年行动计划，设立大健康产业发展基金，争取到全省近70%的医药健康产业专项资金。围绕做强现代中药、做优生物制药、做大化学制药，扶持医药企业实施扩能改造、产品创新、管理创新、人才引进，154个医药健康产业项目开工，申报省医保目录药品175种。举办第二届通化葡萄酒产区高峰论坛，制定山葡萄酒通化产区生产标准，省级葡萄酒产业技术创新战略联盟获批，中国酒庄旅游联盟启动，葡萄酒品牌效应不断提升。出台人参产业健康发展实施意见，益盛汉参等一批亿元以上项目加快推进，新增“长白山人参”品牌原料基地7个，集安人参产业园列入国家现代农业产业园创建名单，人参产业产值突破200亿元。全市开工建设5000万元以上服务业项目172项，100户重点服务业企业实现营业收入65亿元。成立旅游发展委员会，组建文旅发展集团，设立每年1亿元规模的旅游产业发展引导资金，启动全域旅游发展规划编制，出台做大做强冰雪产业实施意见。由吉辽两省12个旅游县（市）组成的“鸭绿江旅游联盟”在集安市成立。太极湾国际旅游度假区、龙堡四季旅游度假区等重点项目进展顺利，滑冰馆开工建设。旅游综合收入增长30.6%。我市入选首批国家中医药健康旅游示范区，被国家旅游局评为厕所革命先进市。参加了央视《魅力中国城》竞演活动，荣获“优秀魅力城市”称号。通化县被认定为全国休闲农业与乡村旅游示范县。省级电商镇和电商村数量分别占全省的25%、23%。

　　（三）农业农村和县域经济稳步发展。深入推进农业供给侧结构性改革，推广粮食绿色增产增效技术面积520万亩，调减籽粒玉米5.5万亩，“旱改水”4万亩，发展鲜食玉米7.5万亩，粮食产量达到36亿斤。永久基本农田划定工作完成，建设高标准农田3.9万亩，耕地保护目标责任制通过省政府考核。实施千万元以上产业化项目20项，农产品加工销售收入增长7%。设施农业、食（药）用菌、林蛙、中小药材等特色产业加快发展，新增棚膜蔬菜面积1.3万亩，新创绿色有机无公害产品标识20个。吉林通化国家农业科技园区通过科技部验收。建设标准化牧业小区40个，畜禽规模化养殖率达到87%。农民专业合作社发展到5834个、家庭农场1114个，土地流转面积132万亩。在全省率先开展新农村建设整市推进工作，570个行政村达标，建成省级美丽乡村14个、美丽庭院1.4万个、干净人家8000户，完成农村改厕1.4万户，全省新农村建设现场会在我市召开。实施水利重点工程49项，水安全和水生态环境得到有效治理。建成农村公路157公里，改造县乡危桥14座。在全省县域综合发展考评中，通化县、辉南县、柳河县分别位列全省一类县第9名、第10名、第12名，集安市位列全省二类县第4名，我市获组织推动工作先进单位。通化县被评为全国首批农业可持续发展试验示范区；柳河县荣获中国火山岩稻米之乡称号，被评为全国首批“四好农村公路”示范县。清河镇入选第二批全国特色小镇，三源浦镇、西江镇、金厂镇入选全省首批特色小镇。

　　（四）向南开放窗口建设步伐加快。交通基础设施建设取得新进展，通集、辉白高速公路加快建设，集桓高速公路纳入全省交通“十三五”规划；高铁站选址已确定，通化火车站整体搬迁改造列入日程，通化至沈阳、至白河高铁前期工作扎实推进；机场扩建列入国家民航“十三五”规划，新增通化至青岛航线，通化机场年旅客吞吐量突破10万人次。开放平台建设取得新成果，国际内陆港务区公路集装箱中心站投入使用，保税物流中心（B型）主体封闭，货物吞吐量突破500万吨。出台支持医药高新区发展意见，开工5000万元以上项目65项，一批重大项目的支撑作用日益增强，产业集群发展格局基本形成。医药高新区被国家科技部认定为创新型企业集群试点、区域性科技服务试点。集安边境经济合作区建设加快推进。成功举办第十一届东北亚博览会-中国通化·长白山国际医药健康产业发展论坛。白通丹经济带发展规划稳步实施；与浙江台州签订合作框架协议，对口合作全面启动。

　　（五）改革创新深入推进。大力实施供给侧结构性改革，压减炼铁产能80万吨，市本级提前完成商品房去库存三年任务。建立债务风险预警机制，争取省政府债券置换资金8.29亿元。落实企业减税、退税政策17.5亿元。清理行政事业性收费和政府性基金、政府定价经营性收费。积极引入社会资本，污水处理厂、卫校升级扩建等4个PPP 项目顺利实施。强力推进“放管服”改革，成立改革协调办公室，出台5个方面、25条具体措施，再次向市辖区下放、委托、授权管理权限106项；政府部门行政职权数量、行政审批项目分别减少44%、30%，非行政许可项目实现“零”审批，部门和项目进厅率均达到100%；梳理行政许可事项清单329项，首批公布政务服务“最多跑一次”事项157项；“多证合一”登记制度改革全面实施，企业登记实现全程电子化，“双随机一公开”监管全覆盖。深化国地税合作，被评为全国百佳国税地税合作市级示范区。企业功能分类、三供一业、公司制改革等国有企业改革不断深入，农村土地确权登记颁证、农村集体产权制度改革试点等工作稳步推进，国有林场改革主体任务基本完成，集体林权制度改革受到国家表彰。财税、事业单位、户籍等改革有序开展。抓好科技创新，列入省科技发展计划项目31项、省校共建计划项目9项，新增国家高新技术企业5户，纳入首批省科技小巨人企业13户，获得省科学技术奖10项，评选表彰市科技进步奖40项。大力推进人才创新，制定了支持人才发展等一系列政策措施，实施“千名学子归巢计划”，开展农村返乡创业人才和“通化工匠”评选活动。集安市、辉南县列入国家级支持农民工等人员返乡创业试点县。

　　（六）城市建管水平明显提升。《通化市土地利用总体规划调整完善方案》通过省政府批准，都市区规划编制完成，浑江百里生态长廊等专项规划和城市综合交通体系规划、总体城市设计有序推进。市区改造棚户区65万平方米，货币化安置棚改户6282套，收储土地114.3公顷，招拍挂出让土地76.6公顷。建设大街、西昌互通立交和官道岭大街主体工程建成通车，红旗桥立交改造全面完工，通化大街升级改造、地下综合管廊、智能立体停车场、绿化美化亮化等重点工程完成年度任务。新建改造燃气管网38.6公里、供水管网34.2公里，新增燃气用户4079户、自来水用户4000户。城市二次供水改造工程获“中国人居环境范例奖”。调峰热源环保提升改造工程完工，新建换热站5座，改造供热管网60公里。启动城市管理体制改革，市级物业管理、环卫管理和城管支队管理权限全部下放。开展城市环境综合整治专项行动，拆除违章建筑7.3万平方米。“五城联创”首战告捷，国家森林城市命名授牌。创建交通管理模范城市通过省级验收，“车让人先行”的文明交通理念深入人心。“智慧城市”建设有序实施，智慧交通、智慧税务建设取得新成效，市应急联动指挥中心一期工程建成使用。荣获“2017中国智慧城市示范城市奖”。

　　（七）生态环境建设力度加大。认真落实中央环保督察部署要求，接受办理信访案件36批、400件，一批生态环保突出问题得到解决，中央环保督察组和省工作协调联络组对我市工作给予高度评价。被评为中国绿色发展优秀城市，获得全省环保目标责任制考核第1名。打好“蓝天保卫战”，淘汰黄标车4302辆，市区淘汰燃煤小锅炉146台，城区空气环境质量优良天数达到322天。实施重点流域污染防治项目10项，市污水处理厂提标扩建完成土建工程，二道江区污水处理厂、浑江城区段清淤和玉带河、柳条沟河等黑臭水体治理完工，重点流域饮用水源水质优良率达到100%。垃圾焚烧发电工程竣工。河长制组织体系全部建立。开展清洁土壤行动，治理水土流失面积160平方公里，滨江西路南段、北出口关闭采石场等地质灾害防治和矿山地质环境恢复治理项目竣工。国家级自然保护区、森林公园、湿地公园生态环境综合整治取得较好成效。实现连续63年无重大森林火灾。集安市被评为国家卫生城市，通化县被评为全国首批生态文明建设示范县。

　　（八）社会各项事业协调发展。义务教育基本均衡发展全部通过国家验收，新增奖补幼儿园29所，市一中新建教学楼投入使用，市中小学示范性综合实践基地二期工程竣工，改造农村薄弱校舍5.4万平方米，创建全国特色校园13所、省特色校园22所，长春中医药大学通化教学区启动建设。城市公立医院改革全面完成，在全省综合考评中位列前茅；代表吉林省接受国家基本公共卫生服务考核，综合得分位列东北三省第1名。建成中医馆71个，基本实现中医药服务人人享有；市中心医院晋级国家三级甲等医院，市人民医院综合门诊楼竣工；在全省率先实现居民健康卡发放与应用全覆盖。文化惠民形式多样、丰富多彩，建成文化大院、小广场13个，基层文化资源覆盖率达到90%以上，万发拨子遗址民俗公园建成开园，人民群众的文化获得感显著提升。话剧《回家·宝贝》获第十五届中国人口文化奖，蒋川蒙目棋吉尼斯世界纪录挑战赛在辉南县举行。成功举办通化市2017年田径运动会，在亚洲少年田径锦标赛、全国跳台高山滑雪等国家级以上赛事中，取得13金6银8铜的优异成绩，提高了通化的知名度。市体育运动学校被命名为“国家高水平体育后备人才基地”，市冬季运动中心被评为全国体育先进单位。民族团结进步和妇女儿童、广播电视、老龄、慈善、住房公积金管理等工作全面发展。

　　（九）民生事业持续改善。城乡常住居民人均可支配收入分别达到25125元、11421元。百件民生实事按期完成。认真落实省委扶贫领域专项巡视反馈意见，严格精准识别、精准退出，坚持不懈整改提升，实施脱贫项目385项，实现7496人脱贫、27个贫困村退出，贫困人口大病报销比例提高到90%以上。城镇新增就业4万人，城镇登记失业率控制在3.95%以内，农村劳动力转移就业24.1万人。社会保障“一卡通”稳步实施。退休养老金月人均增加123元、达到2016元，城镇居民基本医疗补助标准、新农合筹资标准、城镇低保月标准和农村低保年标准分别提高到450元、630元、530元、3600元。改造农村危房8610户，市区1.3万户住房困难家庭享受租赁补贴；出台失能人员长期护理保险制度，5.2万残疾人享受生活困难补贴、护理补贴；资助困难学生2.6万人次。实施农村饮水安全巩固提升工程，解决2.3万人饮水安全问题，贫困村安全饮水实现全覆盖。出台加快养老服务业发展实施意见，依法取缔非法养老机构53家，建成农村养老服务大院8个，老年医院、医疗养老关爱护理中心等项目完工。

　　（十）政风建设和公共服务管理不断加强。全面落实市委关于中央巡视组“回头看”反馈意见整改方案，制定问题清单，建立整改台账，逐条认领、逐项砸实，整改工作取得阶段性成果。深入推进依法行政，加快法治政府建设。提请市人大常委会审议通过《通化市燃放烟花爆竹安全管理条例》、经省人大常委会批准公布，《通化市城乡规划管理条例》有效实施。办结人大代表建议59件、政协提案139件。市长公开电话受理群众来电7.2万件，办复率达到100%。公务用车改革顺利完成。“七五”普法扎实推进，司法行政服务企业、服务项目成效明显，成功创建全国首批税收普法教育示范基地。加强和改进信访工作，一批信访积案集中化解。认真做好十九大期间的安保维稳工作，受到省委、省政府通报表扬。深入开展“安全生产责任深化年”活动，被评为全国“安全生产月”和“安全万里行”活动先进单位。深入实施食品药品放心工程，各县（市、区）全部创建为省级食品药品安全示范县（市、区）。狠抓社会治安综合治理，巡防警务建设成效显著。成功侦破“6·10”特大电信网络诈骗案。“三道防线”加快建设，公安检查站在全省率先建成使用，边境保持安全稳定。国防动员和双拥共建扎实推进。国家安全、外事侨务、气象、人防、地震监测等工作都取得了新成绩。

　　这些成绩的取得，是市委总揽全局、科学决策的结果，是市人大、市政协有效监督、大力支持的结果，是全市人民和衷共济、奋力拼搏的结果。在此，我代表市政府，向全市人民，向人大代表、政协委员，向各民主党派、工商联和无党派人士、人民团体，向驻通国省属企事业单位、驻通部队、武警官兵，向所有关心、支持通化发展的同志们、朋友们，表示衷心的感谢！

　　在总结成绩的同时，我们也清醒地认识到存在的问题：经济发展依然面临较大压力，发展质量和效益还不高，发展不平衡、不充分，产业结构不优、创新能力不强、经济增长缺乏后劲仍然是面临的主要矛盾；实体经济发展困难较多，融资难、成本高等制约企业发展的瓶颈依然存在，投资、营商、人才等环境有待进一步改善；改革开放还要进一步深化，一些重点领域的改革任务还没有落实到位，开放平台作用未能充分发挥，开放引领效应尚未充分体现；财政收支矛盾日益突出；城市功能品质、生态环境保护、保障改善民生、维护社会稳定等方面的任务还很重，与广大人民群众期待还有一定差距；深入解放思想、转变政府职能、根除“四风”顽疾、提高工作质量和效率还需持续用力、久久为功，政府公职人员违法违纪案件仍有发生。对这些问题，我们一定采取有效措施，切实加以解决。

　　二、2018年工作安排

　　2018年，是全面落实党的十九大精神开局之年，是实施“十三五”规划承上启下之年，是通化实现全面振兴发展关键之年，做好今年政府各项工作意义重大。当前，我国经济已由高速增长阶段转向高质量发展阶段，正处在转变发展方式、优化经济结构、转换增长动力的攻关期，必须推动经济发展质量变革、效率变革、动力变革。使命呼唤担当，使命引领未来。我们要抓住机遇，咬定目标，奋力拼搏，攻坚克难，努力夺取新胜利。

　　政府工作的总体要求是：高举习近平新时代中国特色社会主义思想伟大旗帜，更加紧密地团结在以习近平同志为核心的党中央周围，深入学习贯彻党的十九大精神，统筹推进“五位一体”总体布局，协调推进“四个全面”战略布局，按照省十一次党代会、省委十一届二次全会和市七次党代会、市委七届四次全会部署，深入实施“五六七”发展战略，牢固树立新发展理念，牢牢把握稳中求进工作总基调，以提高发展质量和效益为中心，以深化供给侧结构性改革为主线，在发展中保障和改善民生，决胜全面建成小康社会，加快建设吉林省绿色转型发展示范区、东北东部中心城市和国际医药健康名城，奋力开创新时代通化绿色转型、全面振兴新局面。

　　预期目标是：地区生产总值增长6%左右，规模以上工业总产值增长7%左右，地方级财政收入按可比口径与上年持平，城镇登记失业率控制在4.5%以内，城乡常住居民人均可支配收入与经济发展基本同步增长。重点做好以下工作：

　　（一）全力以赴推进项目建设和招商引资。实施5000万元以上项目260项，其中亿元以上项目190项、10亿元以上项目40项。开工建设大禹制药、长海药业、阿尔凯斯特高端精密铸件生产线、海汇龙洲钾长石综合利用等一批新项目，加快推进东宝生物医药产业园、东方红西洋参产业基地、海通现代医药产业园等一批续建项目，推动修正医药科技产业园、万通盛泰生物工程、紫鑫药业异地新建、化工股份技术改造等一批重点项目投产达效。紧盯国家和省产业政策、投资导向，围绕产业发展、基础设施、生态环保、科技创新、改善民生“五大板块”，谋划包装一批投资规模大、带动作用强的大项目、好项目，积极培育中小项目，为保持经济稳健发展积蓄后劲。坚持引资与引智并重，积极有效搭平台、借平台，开展务实精准招商。筹备参加泛珠三角区域合作峰会等系列“吉林行”经贸合作交流，谋划组织国内重点区域和城市商会走进通化等系列招商活动，力争实现招商引资项目履约率90%以上、引资到位率50%以上。继续开展“三抓”“三早”行动，实行5000万元以上项目清单管理，落实项目市级领导包保责任制，推动项目落地开工、达产达效。

　　（二）千方百计稳工业兴产业。重点扶持实体经济高质量发展。开展工业企业提质增效活动，抓好120项重点工业和技术改造项目、10种新产品规模化生产，加强银企对接、产需衔接和生产要素供应保障，扩大药品、钢材、建材等地产品互采互用，确保工业稳定增长。全力支持通钢债转股，加快冶金渣处理、余热发电等节能环保项目建设，推动通钢调整产品结构、转型升级发展。深入推进大健康产业5年行动计划，滚动实施超2000亿元的产业集群项目。制定扶持政策，推动医药企业并购重组，做好新产品研发、重点品种技术升级和大品种二次开发、药品一致性评价，积极培育创新主导型企业，促进医药产业高端化、集群化、规模化发展。大力发展绿色食品产业，实施美好食品、万树保健品等25个亿元以上项目，提高食品产业精深加工能力和附加值。持续推进葡萄酒、人参产业振兴计划，谋划实施葡萄酒小镇，加快建设集安人参国家现代农业产业园等重点项目，开发葡萄酒、人参制品系列产品，擦亮“中国葡萄酒城”和“中国人参之乡”城市名片。抓好玄武岩、石墨、研磨材料、白炭黑等新产品开发、技术改造，促进新材料产业发展。实行市级领导和部门联系民营企业制度，落实支持民营经济发展各项改革措施，开展中小企业入规升级专项行动，组织“企业家节”等系列活动，激发民营经济发展活力。

　　继续打好服务业发展攻坚战，优先发展生产性服务业，积极发展生活性服务业，努力在中高端消费和创新引领方面培育服务业新增长点，不断补齐发展短板。推进全域旅游，做好全域旅游规划编制，制定国家全域旅游示范区创建方案，加快推进金厂养生度假区、永欣水上乐园、游客服务中心等项目建设，进一步提升全域旅游发展水平。加大旅游宣传营销力度，持续开展人参文化旅游节、国际旅游枫叶节、冰雪旅游季等节庆活动，提升“中国通化·康养天下”城市品牌的知名度、美誉度，努力打造国际大健康养生旅游目的地。集安高句丽文物古迹和辉南龙湾生态旅游景区力争晋升为5A级。实施服务业限上企业培育工程，鼓励发展养老、文化、信息等新型消费业态，新增限上商贸企业40户。开展“放心消费”创建活动，打造安全放心的消费环境。扩大对外经贸合作，扶持和鼓励企业积极开拓国际市场。抓好万达城市综合体、玖盛源文化科技研发中心等项目，加快发展电子商务等现代服务业。开展第四次全国经济普查。

　　（三）坚定不移实施乡村振兴战略。按照产业兴旺、生态宜居、乡风文明、治理有效、生活富裕的总体要求，科学制定乡村振兴战略规划，健全城乡融合发展体制机制，优化农业产业体系、生产体系、经营体系，加快推进农业农村现代化。深入推进农业供给侧结构性改革，坚持质量兴农、绿色兴农，促进农业由增产向提质转变。调减籽粒玉米4万亩，建设高标准农田5.5万亩，新创绿色有机无公害产品标识20个。围绕长白山特色产业优势区建设，抓好绿优米、食（药）用菌、中小药材、林蛙、大榛子等特色产业发展，加快康元生物、禾韵蓝莓等34个项目建设，农产品加工业销售收入突破600亿元。围绕建设优质安全畜禽产品基地，继续抓好无疫区建设，新建标准化牧业小区20个。加快农特产业园区、田园综合体、休闲农庄和乡村民宿建设，积极发展休闲农业和乡村旅游。支持家庭农场、农民专业合作社等新型农业经营主体适度规模经营，提高农业全要素生产率。加快农村电商发展，完善农村市场营销网络。围绕江河堤防整治、中小河流治理，实施重点水利工程28项。高标准完成新农村建设整市推进工作，开展农村人居环境整治三年行动，全面推行农村生活垃圾和污水治理，大力推进厕所革命，建设宜居乡村。创建省级美丽乡村10个，改造农村厕所1.4万户。加快培育特色小镇，推进金川旅游、清河人参、西江贡米、光华蓝莓等特色小镇建设。

　　（四）蹄疾步稳推进改革创新。突出供给侧结构性改革这条主线，落实“三去一降一补”任务，做好通钢转型升级和职工安置，巩固商品房去库存成果，完成煤炭去产能任务。深推“放管服”改革，充分借鉴浙江台州“最多跑一次”模式，推进权责清单标准化，推行“互联网+政务服务”“一窗受理、集成服务”，开展“区县同效”试点，推动更多事项“零跑动”办理，抓好“多证合一”“证照分离”改革，“双随机一公开”综合监管实现常态化、标准化、智能化，着力打造一流营商环境。深化金融体制改革，推进金融资源市场化配置，推动企业主板上市和在“新三板”挂牌融资，加强诚信体系建设，让金融更好服务实体经济。吉林省联合股权交易中心通化运营中心力争投入使用。抓好大健康、旅游、人参等产业基金运营管理，加快推进基金实质性运作和项目实施。支持信通、城投等国有企业转型升级，确保国有资产保值增值。加大财政金融风险防控力度，合理控制政府债务规模，坚决守住不发生系统性、区域性风险的底线。做好农村土地“三权分置”和农村集体产权制度改革，土地流转面积占耕地面积的29%以上。深化国有企业、财税体制、商事制度等重点领域改革。

　　深入实施创新驱动战略，争取列入国家、省科技支持项目50项，实施科技小巨人育成计划，省级高新技术企业达到43户。推进与“十四校三所一院”深度合作，推动东宝、万通、康元等质量检测和科技研发中心建设，组建医药健康产业创新联盟，促进重大科技成果转化。稳步提高科研经费投入占GDP比重。加强人才队伍建设，深入实施“千名学子归巢计划”，积极回引通化籍域外人才，大力引进高层次人才，培养高素质技能型人才。

　　（五）持之以恒加快向南开放窗口建设。全面落实省委、省政府大开放战略部署，突出抓好向南开放窗口建设，着力打造全省国际物流新枢纽、开放发展新引擎、绿色转型发展新高地。推进开放通道建设。加快通集高速公路建设，确保辉白高速公路竣工通车，做好集桓高速公路前期工作。抓好通化至沈阳、至白河高铁和通化综合客运枢纽、既有通化站外迁准备工作，加大通集、通丹高铁和机场扩建的谋划推进力度，积极开发培育新航线。推进开放平台建设。推动通化国际内陆港务区引进战略投资者参与建设和运营，争取临时口岸代码和保税物流中心（B型）指标，推进铁路集装箱中心站建设。支持医药高新区跨越发展，谋划建立医药高新区省市共建机制，推动“三园、两区、一中心”建设，实施5000万元以上项目66项，引进亿元以上项目30项，促进产业集群集聚、体制机制和科技创新，扩大产业规模，提升发展效益。加强区域合作，全面落实通丹两市战略合作协议，推动通关一体化、旅游一体化、通道互联互通等重点项目建设，促进东北东部区域合作。推进与台州互动发展，建立更加紧密的合作关系，努力在交流合作与项目建设上实现新突破。

　　（六）精益求精推动城市提档升级。坚持规划先导，修编城市总体规划，抓好综合交通体系规划、总体城市设计和生态新城设计、城市绿道和慢行系统规划、地下空间开发利用规划编制工作，落实都市区规划，强化城乡规划法定作用。实施生态修复和城市修补“双修”工程，进一步改善城市环境、提升城市功能。抓住国家延续棚改支持政策机遇，牢固树立经营城市理念，强化土地收储，提高政府安置房建设比例，市区改造棚户区30万平方米、3267套，让1.1万居民搬出拥挤脏乱的棚户区、住进配套完善的新家园。综合整治老旧住宅小区30万平方米，使18个小区旧貌换新颜。加快城市道路建设，新建续建南山隧道、建设大街延伸等道路和管廊工程，升级改造街路19条，逐期打通城市“断头路”，建成运营智能停车场3个，科学设置交通信号灯、标志、隔离栏等交通管理设施，全面提升路网通达能力和城市承载能力。加快发展城市公交，更新新能源公交车66辆。实施天然气接入工程，推进调峰储配气源建设，改造室内老旧燃气设施6.4万户、新增燃气用户4000户；积极推进长流取水口上移至桃园水库工程，改建供水管网26.7公里；新建换热站5座，改建供热管网9.5公里，保障和改善人民群众用气、用水、用热需求。实施精品绿化和精美亮化工程，新建改建江南湿地等5处公园、绿地和广场。深化城市管理体制改革，推进城市管理职权重心下移。强化小区物业管理，建立以街道、社区为主体的长效管理机制，努力实现物业管理全覆盖。加大城市环境综合整治力度，抓好环境卫生、户外广告、露天烧烤、占道经营等专项整治行动，对违法建筑“零容忍”，发现一处、拆除一处，着力提升市容市貌、市政设施等管理水平。加快新型“智慧城市”建设，深化城市数字化、网格化管理。扎实推进“五城联创”，巩固提升国家森林城市、交通管理模范城市创建成果，切实抓好国家卫生城市、生态文明示范市创建，确保国家园林城市达标。

　　（七）坚持不懈抓好生态文明建设。牢固树立绿色发展理念，坚持节约资源和保护环境的基本国策，像对待生命一样对待生态环境，实行最严格的生态环境保护制度，坚决打好污染防治攻坚战，让通化的“绿水青山”“冰天雪地”变成造福全市人民的“金山银山”。坚决完成中央环保督察组反馈问题整改，建立健全生态环境保护长效机制，实现环境督查监管常态化、全覆盖。落实清洁空气行动计划，加强空气质量监测预警，建设空气质量自动监测站10个；严格排放准入，抓好冶金、化工等产业技术升级、节能改造，减少工业企业污染物排放；严格执行燃放烟花爆竹安全管理条例，加强废弃物焚烧和扬尘污染治理；抓好秸秆焚烧管控，推进秸秆综合利用。落实清洁水体行动计划，按照水资源保护、水生态修复、水环境治理、水污染防治的要求，深入推行“河长制”，实施鸭绿江、哈泥河等重点水系和城市黑臭水体综合治理，在重点流域建设9个水质自动监测站。开工建设浑江百里生态长廊，完成张家大桥至修正大桥段12.1公里浑江干流和保安河、通天河等10条支流治理，力争建成“一廊三区六景”滨水景观，构建“水清岸绿景美、宜居宜业宜游”的生态环境，留下“一泓碧波穿城过，两岸青山排挞来”的乡愁记忆。落实清洁土壤行动计划，开展土壤风险评估和自然保护区、森林公园、湿地公园等重点生态区域环境综合整治，对保护区重要区域实施生态移民工程。整治水土流失面积120平方公里。推进矿产资源节约和综合利用，加快绿色矿山建设步伐。抓好市区地质灾害和矿山地质环境恢复治理。植树造林3.2万亩，建设省级绿美村屯3个。开展全国第二次污染源普查和第三次土地调查工作。

　　（八）科学统筹各项社会事业发展。优先发展教育事业，深化教育改革，率先启动义务教育优质均衡发展，充实、优化教育资源配置，加快普及高中阶段教育，大力发展符合需求的职业教育，努力办人民满意教育。深化医药卫生体制和医保制度改革，力争使医联体建设、分级诊疗和家庭医生签约服务等工作走在全省前列，基本医疗保险实现“六统一”；扎实开展中医药综合改革示范区建设，争创全国中医药工作先进市；扩大妇幼保健、康复等服务和资源供给，全面落实两孩政策，切实加强重大传染性疾病防控。深入实施文化普惠工程，基层文化资源覆盖率达到95%以上；培树百余名通化文化名人、名家、名师和领军人物，创作精品剧目《人参之路》，打造极具通化特色的中医药文化和葡萄酒文化。建设自安山城遗址公园。建成滑冰馆，规划市跳台滑雪训练中心，备战省第十八届运动会和平昌冬奥会。完成市综合档案馆建设。推动民族团结进步、宗教和谐稳定。发展慈善事业。促进社会组织健康有序发展。保障妇女和未成年人权益。加强国防动员和后备力量建设，巩固双拥共建成果，推进军民深度融合发展。

　　（九）不遗余力保障和改善民生。严格落实脱贫攻坚责任制，坚持精准扶贫、精准脱贫，开展“志智双扶”，激发贫困人口内生动力，发展脱贫产业项目，改善贫困区域基础设施条件，确保6400人、28个贫困村实现脱贫。扎实推进就业创业，抓好大学生创业园、农民工返乡创业基地建设。城镇新增就业3万人，农村劳动力转移就业19万人。保护农民工合法权益，农民工工资当期支付率达到100%。实施全民参保登记计划，推进社保扩面和保费征缴，按政策提高基本养老金、失业保险金、城镇医保、新农合补助标准和低保对象大病救助标准。为市区1万户低收入住房困难家庭发放租赁补贴。改造农村危房7233户。扩大困难群众法律援助覆盖面。扎实推进医养融合发展，鼓励支持社会力量兴办养老服务机构，建立居家养老等服务体系，进一步提升养老服务水平，争创全国医养结合示范区。

　　全力维护社会稳定。深化安全生产领域改革，健全安全生产长效机制，落实主体责任，持续开展生产安全、消防安全、道路交通安全等专项整治，整改隐患、不留死角。加强食品药品安全监管，保障群众饮食用药安全。加大信访工作力度，推进信访制度改革，实施依法信访和诉访分离，切实解决群众的合理诉求，促进信访事项及时就地化解。抓好公共法律服务平台建设，创建国家级法治城市。深入推进社会治安防控体系建设，依法打击各类违法犯罪，建设平安通化。完善应急管理协调机制，抓好防汛抗旱、森林防火、地震防灾减灾、人民防空等工作。强化党政军警民合力治边，加大边防基础设施建设力度，维护边境稳定和国家安全。

　　三、全面加强政府自身建设

　　新时代、新目标、新任务，对政府自身建设提出新要求。我们要深入学习贯彻党的十九大精神，牢固树立“四个意识”，增强“四个自信”，持续深入改进作风，持续增强政府执政能力。

　　一是坚持依法行政。谋事做事，须“法”字当头。坚持依法行政、依法办事，法定职责必须为、法无授权不可为。自觉接受人大法律监督、工作监督和政协民主监督，主动接受群众监督和媒体监督。把公众参与、专家论证、风险评估、合法性审查和集体讨论作为重大决策的必经程序，自觉做到在监督的环境下工作，在法治的轨道上尽责，努力建设法治政府。

　　二是坚持为民执政。民之所望，施政所向。把为人民谋福祉作为工作的出发点和落脚点，心往基层想、人往基层走，多谋民生之利、多解民生之忧，多为群众办好事、办实事，始终做到为人民负责，努力建设为民政府。

　　三是坚持实干勤政。“政者，口言之，身必行之”。我们要牢记使命，抓铁有痕，踏石留印，努力把勤奋的精神、实干的劲头、开拓的勇气、自省的作风、有为的追求贯穿工作始终，勤政为民。严肃整治懒政怠政、为官不为，严格督查问责，坚持钉钉子、下绣花功夫，努力建设服务政府。

　　四是坚持廉洁从政。落实全面从严治党要求，把党风廉政建设和反腐败工作不断引向深入。严格遵守中央八项规定实施细则和省委、市委具体规定，廉洁自律，干净干事，坚决防止和纠正各种“四风”新问题。坚持严格要求、严格管理、严格监督，严肃查处各种不正之风和腐败问题，努力建设清廉政府。

　　各位代表，奋斗成就梦想，实干才能兴邦。让我们在市委的坚强领导下，不忘初心、牢记使命，勇于担当、砥砺前行，为开创新时代通化绿色转型、全面振兴新局面而努力奋斗！
